# Research Progress on the Application of Covalent Organic Framework Nanozymes in Analytical Chemistry

**DOI:** 10.3390/bios14040163

**Published:** 2024-03-29

**Authors:** Dongmei Yao, Ling Xia, Gongke Li

**Affiliations:** 1 School of Chemistry, Sun Yat-Sen University, Guangzhou 510006, China; yaodm@hcnu.edu.cn (D.Y.); xialing@mail.sysu.edu.cn (L.X.); 2 Guangxi Key Laboratory of Sericulture Ecology and Applied Intelligent Technology, Hechi University, Hechi 546300, China

**Keywords:** nanozyme, covalent organic framework, analysis, application

## Abstract

Covalent organic frameworks (COFs) are porous crystals that have high designability and great potential in designing, encapsulating, and immobilizing nanozymes. COF nanozymes have also attracted extensive attention in analyte sensing and detection because of their abundant active sites, high enzyme-carrying capacity, and significantly improved stability. In this paper, we classify COF nanozymes into three types and review their characteristics and advantages. Then, the synthesis methods of these COF nanozymes are introduced, and their performances are compared in a list. Finally, the applications of COF nanozymes in environmental analysis, food analysis, medicine analysis, disease diagnosis, and treatment are reviewed. Furthermore, we also discuss the application prospects of COF nanozymes and the challenges they face.

## 1. Introduction

An enzyme is a kind of protein or RNA that has high specificity and good catalytic activity for substrates, which is an indispensable substance for all living things [1]. It achieves the purpose of increasing the catalytic reaction rate by specifically reducing activation energy [2]. However, most natural enzymes are difficult to prepare, and they need to be used in mild environments because they easily lose activity in hot, acid, alkali, and other environments. These shortcomings limit the development of natural enzymes. Therefore, scientists have been exploring ways to synthesize artificial enzymes in vitro and have made a series of progress. An artificial enzyme has the advantages of simple preparation and stable performance and has huge application prospects in biochemistry [3]. With the rise and development of nanotechnology, new ways have been provided for the study of nanoparticle artificial enzymes with enzyme-like activity, and the concept of nanozymes also rises.

In 2007, magnetic nanoparticles Fe_3_O_4_ were found to have the same catalytic performance as horseradish peroxidase, which can catalyze H_2_O_2_ to oxidize various horseradish peroxidase substrates [4]. Based on the research results, they proposed that Fe_3_O_4_ nanoparticle is a kind of peroxidase mimetic enzyme. The appearance of nanozymes has also attracted many people’s attention. Nanozymes have good stability, low cost, and high catalytic efficiency and have been used in environmental, chemical, food, and other fields [5]. Since the discovery of nanozymes, this has broadened the application scope for materials science, physics, and biology, and at the same time, endowed nanomaterials with new characteristics and enriched the content of artificially simulated enzymes. As a new kind of mimic enzyme, nanozymes have developed rapidly and become an excellent alternative to natural enzymes and have gradually expanded into a new research direction [6]. Currently, a wide variety of reported nanozymes includes metal nanoparticles [7], metal oxide nanoparticles [8], carbon nanomaterials [9,10,11], and so on. According to structural characteristics, nanomaterials can be classified into three categories, namely iron-based nanozymes, non-ferrous metal nanozymes, and carbon nanozymes. Among them, carbon dots, graphene, and organic framework materials are all carbon nanozymes, and research on their enzyme-like activities has also been reported [12,13]. Generally, metal-based nanozymes have poor selectivity and are prone to aggregation, resulting in a decrease in enzyme activity [14]. Covalent organic framework (COF) ligands can cleverly mimic the active sites of natural enzymes and can greatly improve the above problems. Therefore, the design and application of COF nanozymes have aroused great interest.

In 2005, Cote et al. [15] first reported COF, which is a porous crystalline nanomaterial composed of symmetric and rigid organic structural units. COFs have received widespread attention due to their ordered channel structure, excellent storage and catalytic properties, and easy modification [16,17]. At the same time, COFs are also a type of new carbon-based nanozymes. Compared with other nanozymes, COFs can design the structure accurately, so they can control the specific functional structure by chemical means [18]. Researchers can control the selectivity and catalytic activity of COF nanozymes to the substrate by controlling the nano-effect of COFs. Based on this, the research on COF nanozymes has gradually aroused people’s interest. Based on the structural diversity and adjustability of COFs, COFs have been developed as nanozymes that catalyze various substrates. Recently, the application of COFs in analysis has developed rapidly, and there are many reviews related to COFs, including their preparation and applications. However, in most reviews, the role played by COFs is not that of nanozymes. Or even if there are reviews related to COF nanozymes, COF nanozymes are only a small part of the review, and the application of COF nanozymes has not yet fully covered the environment, food, and medicine [19,20]. Therefore, focusing solely on the topic of COF nanozymes, a comprehensive review of their applications in analytical chemistry has not yet been found.

**Figure 1:**
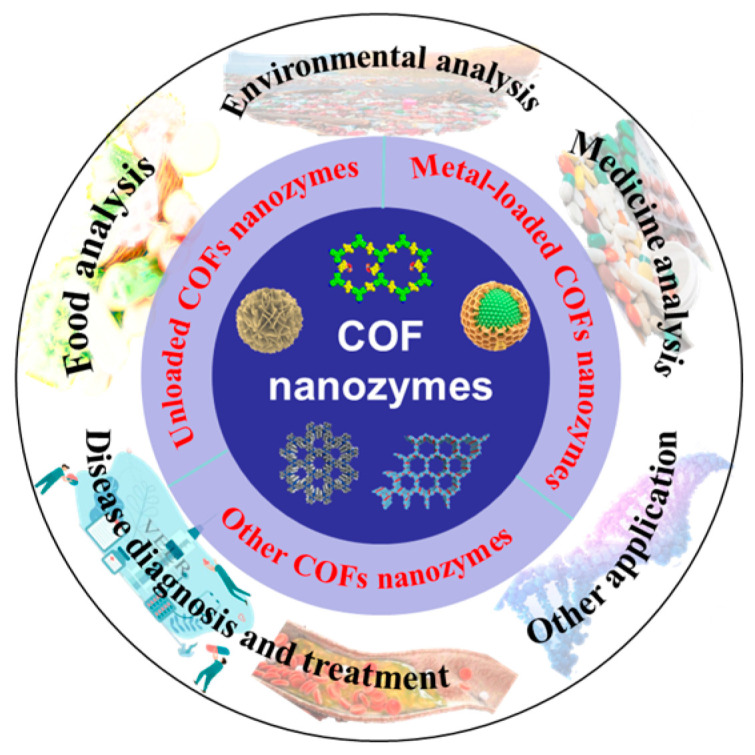
Overview of COF nanozyme applications.

Here, we summarize the synthesis principles, types, and methods of COF nanozymes. At the same time, the applications of COF nanozymes are comprehensively reviewed, including environmental analysis, food analysis, drug analysis, and disease treatment and diagnosis (Figure 1). The prospects and challenges of COF nanozymes are also discussed. We hope that this review can expand ideas for the research of COF nanozymes.

## 2. Synthesis of COF Nanozymes

### 2.1. Design Principle of COF Nanozymes

**Figure 2:**
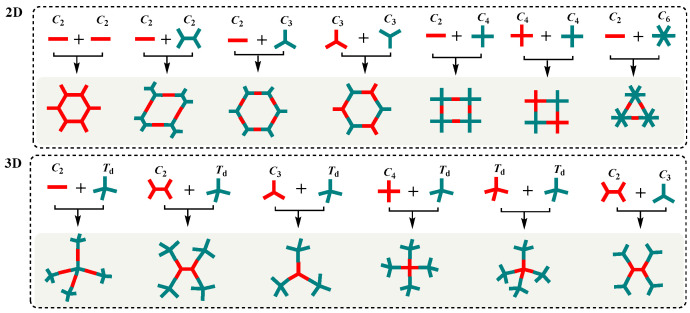
Design schematics for 2D and 3D COFs.

COF is a porous crystalline material with a highly ordered structure, and its pore size, shape, and surface microenvironment can be pre-designed through a topological structure diagram [21,22,23]. According to the topological diagram, there are two types of COFs, 2D and 3D, but the reported COF materials are mainly 2D topological structures [24]. Different combinations of different building units constitute the diversity of COF structures (Figure 2). In COFs, different nodes and connectors are bonded by covalent bonds, forming an ordered periodic layered stacking structure in a two-dimensional plane, and the layers form nano-pores by π-π interaction [25]. The abundant building blocks and connecting bonds ensure the diversification of the COF structure.

**Figure 3:**
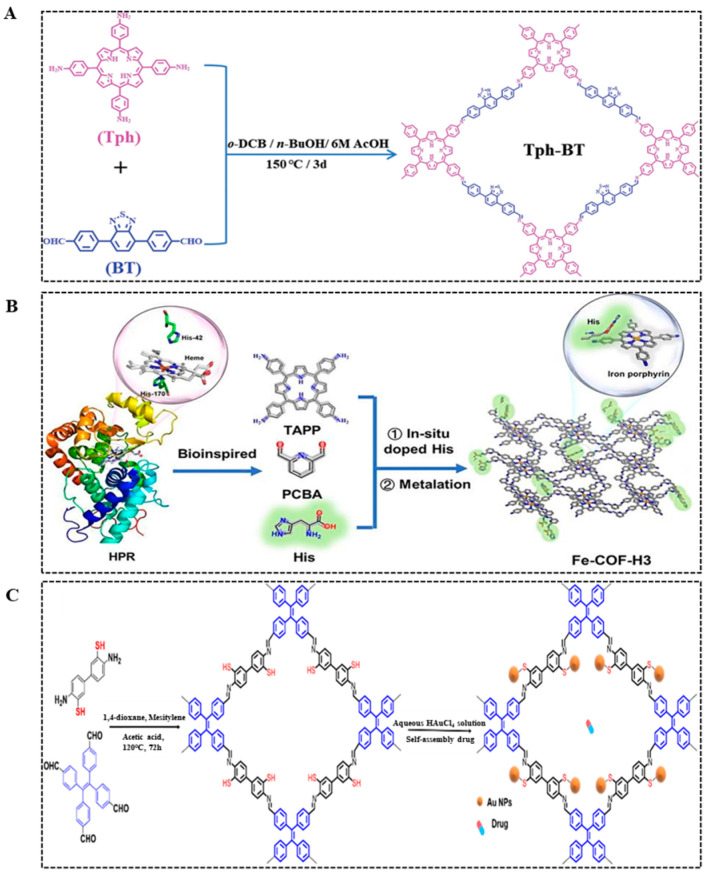
Synthetic pathways of nanozymes: Tph-BDP (**A**) [26], Fe-COF-H3 (**B**) [27], and COF-Au@Cisplatin [30] (**C**).

The surface of COFs has abundant functional groups and active sites, which are potential targets for constructing nanozymes. Zhang et al. [26] used a solvothermal synthesis method to prepare an imine-linked tetragonal COF nanozyme (Tph-BDP) through the combination of [C2 + C4] (Figure 3A). Under the illumination condition of LED light, Tph-BT has high simulated oxidase activity. The enzyme activity of Tph-BDP remains stable between 10–60 °C, and the thermal stability is good. At the same time, COFs have tunable chemical structures and large surface areas. It is also feasible to create COF nanozymes by introducing functional fragments of related natural enzymes into the COFs to mimic the structure of natural enzymes. Zhong et al. [27] synthesized imine-linked COF nanozymes (Fe-COF-H3) using a one-pot method by introducing histidine (His) into the COF structure (Figure 3B). Fe-COF-H3 has strong simulated peroxidase activity, and the in situ introduction of His enhances the catalytic activity of COFs. The practice has proved that the solution of introducing natural enzyme fragments on the surface of COF is feasible.

Currently, designing COF nanozymes by mimicking the structure of natural enzymes is still in the early stages of research. In most COF nanozymes, the main role of COFs is to serve as carriers. COF nanozymes are prepared by loading nanomaterials with rich functional groups in the COF structure. Using the same amines and aldehydes as monomers, the researchers prepared imino-linked COFs [28,29]. Then, COF nanozymes with simulated nitroreductase activity and peroxide-mimetic enzyme activity were prepared by in situ loading AuNPs in COF through hydrogen bonding or π-π bonding. Zhou et al. [30] also used the condensation reaction of aldehydes and amines to prepare imine-linked tetragonal COF nanozymes (Tph-BDP) through the combination of [C2 + C2] and the solvothermal synthesis method. Then, AuNPs were loaded in situ through strong Au-SH interaction to prepare COF nanozymes for the treatment of liver cancer (Figure 3C). From the above research results, it can be concluded that the design of COF nanozymes is traceable.

### 2.2. Types and Characteristics of COF Nanozymes

Based on the various design pathways of COFs, the types and characteristics of COF nanozymes are also diverse. Below, we mainly introduce three types of COF nanozymes: unloaded COF nanozymes, metal-loaded COF nanozymes, and other COF nanozymes.

#### 2.2.1. Unloaded COF Nanozymes

**Figure 4:**
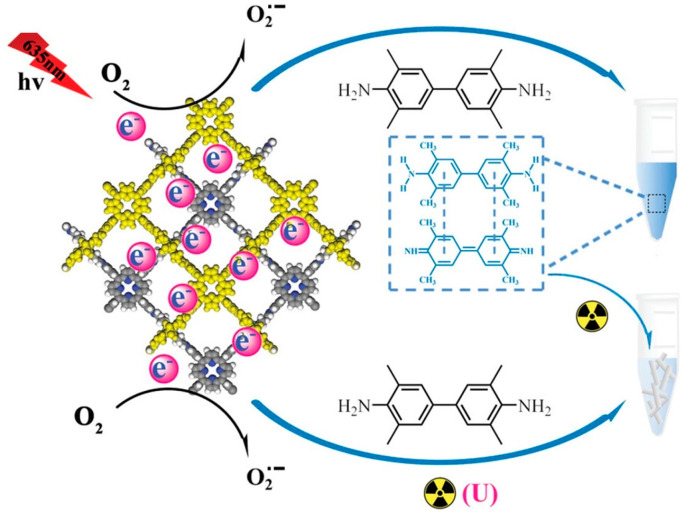
Tph-BDP nanozyme simulates the oxidase process [40].

COFs have a designable pore structure and adjustable porosity, which allow the substrate to effectively access the catalytic activation site and accelerate the mass transfer of the catalytic reaction [31]. Triazine COF is a porous organic framework material, which is usually obtained by trimerization of triazine or nitrile-containing monomers [32,33,34]. In 2008, Kuhn et al. [35] first synthesized triazine COFs through nitrile trimerization using ZnCl_2_ as a catalyst under ionization heat conditions of 400 °C. Triazine COFs can maintain stability at high temperatures and have a large surface area. Based on the excellent properties of triazine COFs, researchers are committed to applying them to the field of nanozymes. A triazine COF (TTA-Tp COF) that has simulated oxidase activity was synthesized and used to rapidly catalyze the oxidation of 3,3′,5,5′-tetramethylbenzidine (TMB) in visible light [36]. Moreover, TTA-Tp COF nanozyme has good stability, excellent catalytic performance, and is easy to be controlled by light. The authors used monomers with different electronic properties to synthesize COF, making it possess good light absorption properties and photoconductivity, thus successfully constructing metal-free COF nanozymes [37,38]. Compared with triazine-based COF nanozymes, imino-COF nanozymes are the focus of researchers. Imine-linked COFs have gradually become the most popular type of COF due to their wide variety of monomers, ease of synthesis, and good stability [39]. Exploring the application of imine-linked COF nanozymes has received increasing attention. Zhang et al. [40] synthesized imine-linked COFs (Tph-BDP) for the first time using porphyrin derivatives as monomers. Under visible light irradiation, Tph-BDP can rapidly catalyze the oxidation of TMB and exhibit excellent simulated oxidase activity (Figure 4).

It is reported that some active sites can also be introduced into the framework structure of COF nanozymes to enhance the catalytic activity [41]. For example, catalytic active sites containing heteroatoms can be introduced into the COF structure to give COFs a certain simulated enzyme activity, such as pyridine, imidazole, or transition metals. Xiao et al. [42] used 2,4,6-Trimethyl-3,5-dicyano-pyridin (DCTP) as the monomer and introduced pyridine groups into the COF framework to prepare DAFB-DCTP COF to possess hydrolysis active sites, giving it the activity of simulating hydrolase. Expanding COFs as a member of nanozymes makes it possible to control the structure and performance of nanozymes. These COF nanozymes do not require loading nanomaterials, and all exhibit excellent simulated enzyme activity, but most of them require experiments under light conditions. This shows that COFs alone have low activity as nanozymes, and further optimization of experimental conditions is required.

#### 2.2.2. Metal-Loaded COF Nanozymes

In addition, the surface microenvironment of COFs is abundant, and the surface area is large, which can allow COFs to obtain well-dispersed active binding sites to efficiently and selectively load nanozymes on the surface of COFs. Among them, COF surface-loaded metal nanoparticles are the most common COF nanozymes. For example, metal-loaded COF nanozymes can be prepared by loading gold nanoparticles or silver nanoparticles on the COF surface [29,30]. Among the many COF carriers, boron-linked COFs have high crystallinity and low density, but they are sensitive to water, resulting in poor stability, which may limit the application of boron-linked COFs in some aspects [43]. Therefore, the construction of more stable COFs in aqueous solutions has attracted the attention of researchers, and imine-linked COFs have also emerged [44,45,46]. Although imine-linked COFs are insensitive to water, the imine-linked reversible linkage is not stable enough. To convert reversible bonds into irreversible bonds and further improve the stability of imine-based COFs, researchers have developed bond conversion strategies [47], bond protection strategies [48], and other methods, which have a profound impact on the structural stability of COFs.

**Figure 5:**
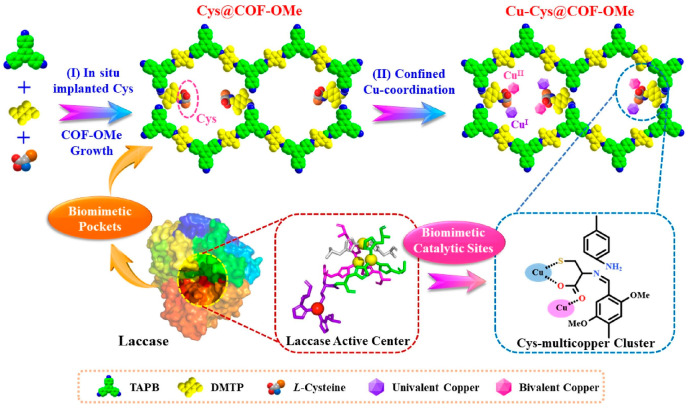
Preparation route of Cu-Cys@COF-OMe namozyme [54].

The condensation reaction of amines and aldehydes can produce imine-based COFs. It was first reported in 2009 by Uribe-Romo et al. using tetrakis (4-phenyl aryl) methane and terephthalaldehyde as monomers and named as COF-300. [49] The COF-300 has a pore diameter of 7.2 Å, a high surface area, and good stability. With the formation of -C=N-, the stability of COF has also been greatly improved [31]. In addition, the imine-linked COF skeleton is rich in nitrogen and can coordinate with metal ions, providing feasibility for the preparation of metal-loaded COF nanozymes [50,51,52]. Imine-linked COFs can be combined with metal materials through Au-SH bonds [30], coordination of unsaturated amino groups [53], and other forces to prepare COF nanozymes. An imine-linked COF (COF-OMe) was reported by Tang et al. [54] After modifying COF-OMe with cysteine, multi-copper clusters (Cu^+^/Cu^2+^) were introduced on the COF surface through copper coordination, and a COF nanozyme with laccase activity (Cu-Cys@COF-OMe) was prepared (Figure 5). Cu-Cys@COF-OMe has an excellent catalytic activity to simulate laccase. It can be seen from the currently reported literature that these metal-loaded COF nanozymes have excellent simulated enzyme activity, have mild reaction conditions, and do not require light excitation. Therefore, imine-linked COF nanozymes have promising applications in the field of analysis.

#### 2.2.3. Other COF Nanozymes

**Figure 6:**
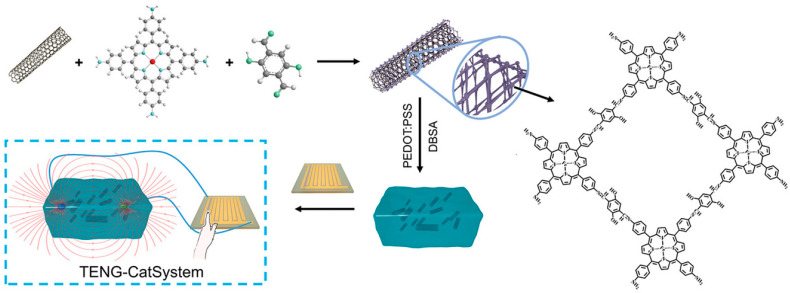
Preparation route of wearable catalytic system TENG-CatSystem [57].

In addition to loading metal materials, COF nanozymes can also load other types of substances, such as carbon nanotubes, metal-organic frameworks (MOFs), natural enzymes, etc. [55]. However, there are relatively few reports. Compounding carbon nanotubes, graphene, etc., with COFs increases the diversity of COF nanozymes and can also complement each other in structure or performance [56], which is an ideal choice for preparing COF nanozymes. Yao et al. [57] coated COF on carbon nanotubes (CNTs) to synthesize COF-CNT nanozymes. Utilizing its peroxidase-like activity, a wearable catalytic system TENG-CatSystem was prepared and applied to cancer treatment (Figure 6). The loading of CNTs enhances electronic conductivity and catalytic performance. MOFs are also important porous nanomaterials, which are generally prepared from metal ions and organic ligands. They have a rich metallic center [58]. Assembling COFs and MOFs into highly dispersed heterostructures is beneficial to improving the catalytic performance of COF nanozymes [59,60]. As a carrier to load MOFs, COFs can not only maintain the structural stability of MOFs but also promote mass transfer [61]. Based on this, researchers began to focus on the study of MOF-COF composite nanozymes. Hou et al. [62] assembled COF on ZIF-8 through the affinity of organisms and synthesized a MOF-COF hybrid nanozyme. MOFs-COFs can rapidly catalyze the oxidation of 4-aminoantipyrine with H_2_O_2_ and exhibit excellent peroxidase-mimetic enzyme activity. At present, the report of MOF-COF composite nanozymes is still in its infancy, but it is not difficult to infer the huge potential of MOF-COF nanozymes from the reported literature [63,64]. It can be seen from the current research progress that loaded COF nanozymes are the focus of research, among which metal-loaded COF nanozymes are the main ones. Among many COF carriers, imino-linked COFs have become the most important COF carriers because of their stronger affinity.

### 2.3. Synthesis Method of COF Nanozymes

In addition to monomers, the morphology of COF nanozymes also depends on the synthesis method and conditions [65]. So far, the main synthesis methods of unloaded COF nanozymes are solvothermal synthesis and microwave-assisted synthesis [66,67]. Solvothermal synthesis is the most popular method for synthesizing COF nanozymes and is also the chief method used to synthesize COFs [15]. The reaction process is carried out in a closed environment, using organic solvents as the medium, and under certain temperature and pressure conditions. Among them, the concentration of the catalyst, reaction time, temperature, and pressure all affect the structure and activity of COF nanozymes. Based on the solvothermal synthesis method, researchers have conducted a lot of work on the performance of COF nanozymes [68,69]. Solvothermal synthesis is widely used and is conducive to the large-scale preparation of COF nanozymes [65].

**Figure 7:**
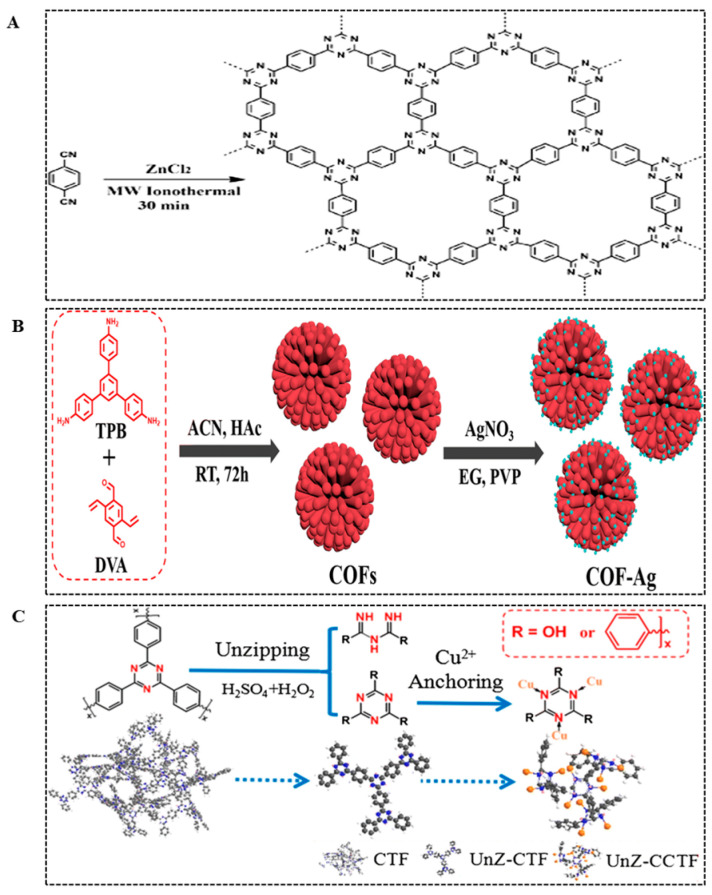
Synthetic methods of CTF nanozyme (**A**) [72], COF-Ag nanozyme (**B**) [73], and UnZ-CCTF nanozyme (**C**) [74].

A microwave is an electromagnetic wave that can selectively heat materials. Compared with traditional heating methods, microwave heating can penetrate the sample and transfer heat faster and more evenly [70]. As an alternative energy source, microwave heating has also attracted the attention of COF nanozyme researchers [71]. He et al. [72] used 1,4-dicyanobenzene as a monomer to synthesize a triazine-based COF (CTF) through the microwave method, and the product can be easily obtained within 30 min (Figure 7A). Compared with solvothermal synthesis, microwave-assisted synthesis has a faster reaction rate and takes less time. In recent years, the use of the microwave method to prepare COF nanozymes has also attracted widespread attention. However, microwave-assisted synthesis also has problems, such as difficult reaction control and large-scale synthesis.

For loaded COF nanozymes, the carriers used are mostly imine-linked COFs, and the preparation method depends on the type of loaded object. Based on the abundant nitrogen element on the surface of imine-linked COFs, the loading of noble metal nanoparticles generally adopts the in situ reduction method of NaBH_4_, citrate, or ethylene glycol [29]. Liu et al. [73] obtained imine-linked COF through a Schiff base reaction and then used ethylene glycol (EG) as the reducing agent to load AgNPs in situ on the COF surface to prepare COF-Ag nanozyme (Figure 7B). In addition, by modifying the surface of COFs with -SH, the loaded noble metal nanoparticles can be reduced in situ with a reducing agent through the affinity of the noble metal with -SH [53]. For some metal ions, coordination can be used for loading [74,75]. Ma et al. [74] prepared triazine COF (CTF) and used the N element of the triazine structure to coordinate Cu^2+^ through Cu-N to prepare COF nanozymes (UnZ-CCTF) (Figure 7C). In addition to the common synthesis methods introduced above, researchers have also successively developed methods such as room-temperature etching and self-assembly methods. Although the research on these methods is not extensive enough, they have also enriched the synthesis pathways of COF nanozymes and provided new ideas for subsequent research.

Based on the classification types of COF nanozymes, the performance of COF nanozymes was compared and listed in Table 1. It can be seen from the results that most COF nanozymes have the performance of simulating oxidases or peroxidases. The main types of COF are imine-linked COF, the synthesis method of unloaded COF nanozymes is mainly solvothermal synthesis, and the main method of synthesis of loaded COF nanozymes is the in situ reduction method or supplemented by ultrasonic conditions. In recent years, the synthesis and application of hybrid COF nanozymes have developed rapidly, and “all-in-one” functional COF nanozymes have become a hot research topic. Some innovative synthesis methods have also emerged. For example, some researchers used unique invisible cell armor to anchor COF to prepare COF nanozymes, which has a great impact on improving the biocompatibility and specific targeting effect of COF nanozymes. Judging from the current research situation, COF nanozymes have broad application prospects, but their performance in simulating natural enzymes needs to be further studied.

## 3. Application of COF Nanozymes in Analytical Chemistry

Recently, COFs have developed rapidly, and at the same time, they also exhibit fascinating enzyme-like activities. The analytical application based on COF nanozymes has gradually attracted the attention of researchers. At present, the analytical methods used in the application of COF nanozymes include colorimetric methods [62], chemiluminescence methods [86], electrochemical methods [87], fluorescence methods [88], etc. Among them, colorimetric methods are the most widely used analysis methods. Next, we will mainly summarize the research progress of COF nanozymes in environmental analysis, food analysis, medicine analysis, and disease diagnosis and treatment.

### 3.1. Environmental Analysis

With the acceleration of industrialization, environmental pollution problems are becoming increasingly serious. For example, water, soil, and air pollution have caused huge harm to human survival and development. Monitoring environmental pollutants is of great significance. Phenolic compounds are important chemical raw materials and can be used to manufacture dyes, drugs, and other products. At the same time, phenolic compounds are toxic substances that are toxic to all living individuals. Their aqueous solutions and vapors can be harmful to humans and cause poisoning. Tang et al. [54] first implanted cysteine in situ into COF and then introduced Cu ions through coordination to prepare a laccase biomimetic nanozyme. The nanozyme can effectively capture phenolic pollutants in water and catalyze the oxidation and degradation of pollutants. Its catalytic activity is high, and the activity of laccase is about 1/2 of it, which provides a good path for the large-scale removal of phenolic pollutants in water. The holes in the COF also serve as biomimetic pockets for laccase, which can improve the capture efficiency of phenolic pollutants and enhance the activity of the nanozyme. In addition, the enzymatic activity of the COF nanozyme under different conditions (such as ionic strength, storage time, temperature, ethanol, pH, etc.) was also investigated. The results show that the COF nanozyme has good catalytic stability and recyclability. This method has high phenol degradation efficiency and good stability and is expected to be used in the treatment of industrial environmental wastewater in the future. To achieve a quantitative analysis of phenolic pollutants, Hou et al. [62] synthesized COF-enhanced simulated peroxidase hybrid nanozyme Fe_3_O_4_@ZIF-8@COF and constructed a colorimetric sensor to detect 6–350 μmol/L phenol in water. Sun et al. [89] also prepared a COF-based electrochemical nanozyme to detect hydroquinone in a water environment. Experiments proved that the COF nanozyme has strong simulated horseradish peroxidase (HRP) activity, and the detection results were satisfactory. The introduction of COFs improves the stability and dispersion of nanozymes and is expected to be used as a case study in practical applications in environmental monitoring stations in the future. 

**Figure 8:**
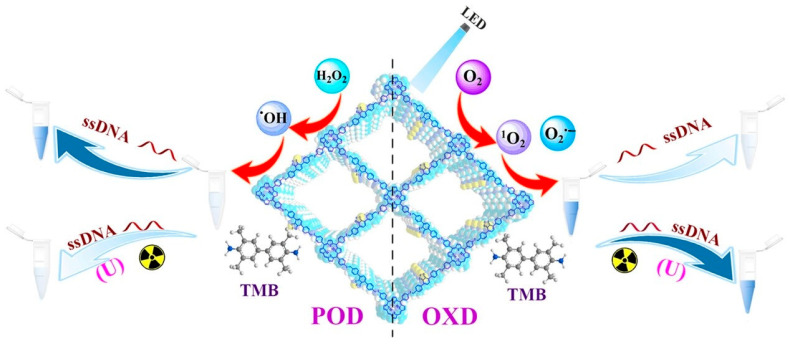
Schematic diagram of UO_2_^2+^ detection [76].

Uranium is a radioactive element that can easily cause cancer and some liver and nervous system diseases after entering the human body. In the environment, it usually exists in the form of uranyl (UO_2_^2+^), which is a highly toxic environmental pollutant [90]. Zhang et al. [40] prepared a COF nanozyme (Tph-BDP) with simulated oxidase activity for the first time. Under visible light conditions of 635 nm, Tph-BDP can catalyze TMB to generate blue oxide. UO_2_^2+^ can coordinate with TMB oxide, causing fading of the solution. Therefore, a colorimetric platform was constructed to detect 0.05 µmol/L UO_2_^2+^. In addition, experimental results also show that Tph-BDP has good thermal stability. On this basis, Zhang et al. [76] continued to develop a photosensitive COF nanozyme (Tph-BT), which has excellent oxidase activity. Single-stranded DNA (ssDNA) and UO_2_^2+^ were introduced to regulate the enzyme activity of Tph-BT, and a colorimetric sensor was constructed to detect UO_2_^2+^ in water (Figure 8). The author effectively utilized the photocatalytic properties of COF, making COF play a huge role in the field of nanozyme analysis. And due to the introduction of ssDNA, the analysis method has better selectivity. Moreover, UO_2_^2+^ can also be used to regulate the catalytic ability of COF nanozyme to establish the colorimetric platform [77]. The introduction of COF nanozymes has expanded the application of environmental analysis and improved the sensitivity of analytical methods. However, these analytical methods are still in the standard laboratory research stage, and further in-depth research is needed to be used as research examples in practical applications in environmental monitoring.

### 3.2. Food Analysis

**Figure 9:**
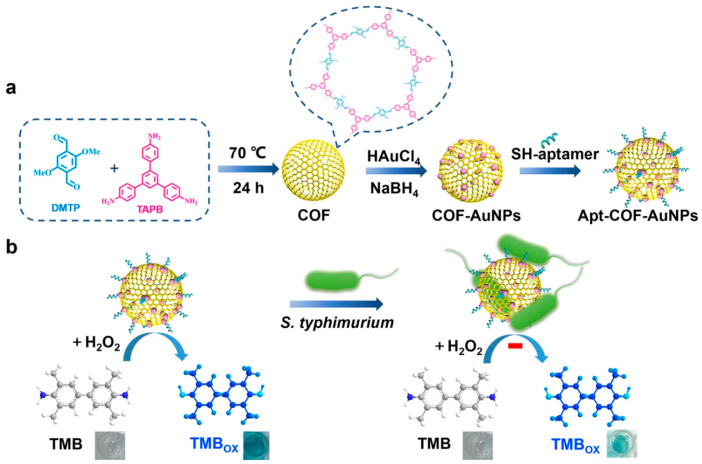
The preparation of COF-AuNP biosensor (**a**) and determination of *Salmonella typhimurium* (**b**) [53].

In recent years, food safety incidents have occurred frequently, causing widespread concern in society. Salmonella is a common foodborne pathogen that affects both humans and animals. Food poisoning may occur through contact with food contaminated with Salmonella, and the probability of poisoning is the highest. *Salmonella typhimurium*, a non-adaptive or pantropic Salmonella, is one of the main pathogenic bacteria causing acute gastroenteritis. Accurate and sensitive identification and monitoring of *Salmonella typhimurium* is important for food safety preparedness. Wei et al. [53] prepared imine-linked COF and then loaded AuNPs on the COF surface to obtain a COF-AuNP composite. COF-AuNPs have strong simulated peroxidase activity. A biosensor (apt-COF-AuNPs) was prepared by modifying aptamers on the surface of COF-AuNPs to detect *Salmonella typhimurium*. The apt-COF-AuNPs have strong catalytic ability for the H_2_O_2_-TMB system. When *Salmonella typhimurium* is present, apt-COF-AuNPs selectively capture *Salmonella typhimurium*, and the catalytic ability of the system is weakened (Figure 9). There is a positive correlation between TMB color change and *Salmonella typhimurium* concentration of 10–10^7^ CFU/mL. Ultimately, this method achieved good sensing results for *Salmonella typhimurium* in water and milk. The presence of spherical COF increased the specific surface area, which can accommodate more AuNPs, thereby improving the enzymatic activity of COF-AuNPs. In this analysis method, the authors introduced advanced smartphone technology into the detection of samples, which plays an important role in on-site testing. In addition, it was also verified that apt-COF-AuNPs have good stability and are expected to be further applied in real life.

Since synthetic organic pesticides were used in agricultural production, they have caused serious pesticide pollution problems and become an important factor endangering life safety. Currently, most of the pesticides used can biodegrade into harmless substances in a short time, but organochlorine pesticides are difficult to degrade. Humans can become poisoned by eating food contaminated with pesticides. Li et al. [80] prepared a PB@Fe-COF@Au nanozyme. PB@Fe-COF@Au has strong simulated peroxidase activity. Acetylthiocholine can be converted to thiocholine in the presence of acetylcholinesterase. Thiocholine will inhibit the activity of PB@Fe-COF@Au, resulting in a lighter color of the TMB-H_2_O_2_ reaction. The presence of organophosphorus pesticides will reduce the production rate of thiocholine, ultimately achieving colorimetric detection of 10–800 ng/mL chlorpyrifos in food. The authors used COF as a shell to encapsulate PBNPs and AuNPs to prepare PB@Fe-COF@Au nanozymes, which improved the dispersion and catalytic activity of the nanoparticles. The authors also experimentally proved that the COF nanozyme has good storage stability in deionized water, which also proved the reliability of the COF nanozyme analysis method. Using COF as a carrier, Ma et al. [86] loaded Candida lipase (CRL) on COF to prepare a COF@CRL nanozyme. The luminol-dissolved oxygen reaction can be catalyzed by COF and produce a chemiluminescence (CL) signal. The CRL can accelerate the hydrolysis of herbicide ether, and the phenols produced will inhibit the CL signal. Based on this, a one-step chemiluminescence method was constructed to detect the 11 nmol/L herbicidal ether content in vegetables and fruits. In addition, the authors experimentally verified the good storage stability of COF-300-AR@CRL and the reproducibility of herbicide determination. This method is very promising for practical applications in food analysis in clinical laboratories. Based on COF nanozymes, Liang et al. [91] also developed a colorimetric sensor to detect the organophosphorus pesticide trichlorfon, and the results were quite satisfactory. The prepared COF (TpBTD) has strong simulated oxidase activity. Different from other COF nanozymes, in this paper, the author triggered the activity of COF nanozymes through flashlight illumination and then used a smartphone to identify colors, which was relatively safer, greener, and more convenient. It has important application significance in on-site monitoring.

Beyond bacteria and pesticides, heavy metal pollution is another key factor causing food problems. Cd^2+^ is a heavy metal ion. When it enters the human body, it can cause heavy metal poisoning and threaten human health. Li et al. [92] prepared a soluble starch-loaded COF (COFTpBD-SS) nanozyme and then controlled its catalytic ability through peptides to construct a SERS sensor to detect 0.025–0.95 nmol/L Cd^2+^ in rice. From the currently reported applications of COF nanozymes in food analysis, it can be seen that using COFs as a carrier to load nanoparticles has broad application prospects.

### 3.3. Medicine Analysis

Drug analysis plays an important role in various aspects of drug research, production, testing, and clinical application. As an important prescription drug, captopril has a unique effect in the treatment of heart failure or high blood pressure [93]. Yuan et al. [36] developed a triazine COF (TTA-Tp COF). TTA-Tp COF has strong simulated oxidase activity. Under LED light irradiation, TTA-Tp COF can catalytically oxidize TMB to oxTMB, and the solution turns blue within 10 min. Captopril (Cap) can effectively reduce the oxidation efficiency of TMB. Therefore, a simple analytical platform to detect 1–100 μmol/L Cap was established. The authors utilized the photosensitive catalysis of TTA-Tp COF itself to realize the detection of captopril. In addition, in the absence of light, COF can also be used as a carrier for nanoparticles to improve enzyme activity by improving the dispersion of nanoparticles. If the method is to be applied clinically, its reliability and reproducibility need to be further studied. Ciprofloxacin is a widely applicable antibacterial drug that can be used for infections caused by bacteria. Li et al. [94] prepared COF (COF-AIECL) with aggregation-induced electrochemiluminescence (ECL) and then modified Fe_3_O_4_@Pt NPs in COF-AIECL as nanozymes. The ECL signal was selectively regulated through the modification of molecularly imprinted polymers, thereby achieving the purpose of detecting 5.98 × 10^−13^ mol/L ciprofloxacin. The detection limit of the method reaches 0.5 pmol/L. It can be seen that the use of COF nanozyme is conducive to improving the sensitivity of the method. In addition, the authors also examined the selectivity, stability, and reproducibility of the analytical method. The experimental results were satisfactory and are expected to be applied in clinical experiments in the future.

**Figure 10:**
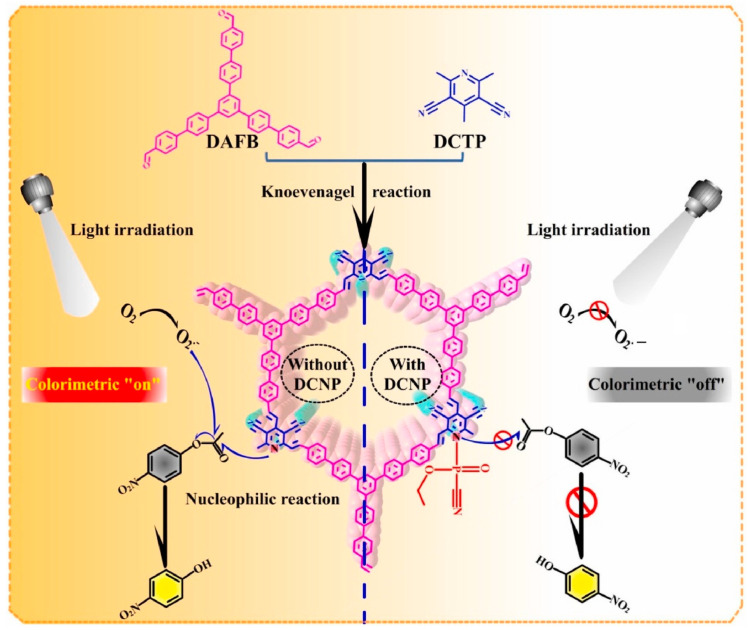
Schematic diagram of DAFB-DCTP COF colorimetric determination of DCNP [42].

Nerve agents are toxic chemicals that destroy the normal conduction function of the nervous system. They can poison people through the respiratory tract, skin, and other channels and are highly lethal. Xiao et al. [42] developed a 2D COF nanozyme, which has strong simulated hydrolase activity and can photo-catalytically degrade the enzyme–substrate p-nitrophenyl acetate. In the presence of diethyl cyanophosphonate (DCNP), the catalytic reaction is inhibited. Therefore, a colorimetric platform to detect DCNP was established (Figure 10). The COF nanozyme showed high stability in either light or alkaline solution. Judging from current research results, most COFs have enzyme-like activity under the action of light. If COFs are used as carriers and combined with nanozymes to prepare complexes, most complex nanozymes do not require light. Analytical methods based on COF nanozymes have great prospects for practical application in medicine analysis.

### 3.4. Disease Diagnosis and Treatment

Cancer is a collective name for a series of malignant tumors that grow rapidly, are highly invasive, and are extremely harmful to the human body. Among many major diseases that seriously threaten human life safety, cancer is plaguing mankind due to its high mortality rate and extremely low cure rate. To reduce cancer mortality, it is important to develop accurate early diagnosis technology. Sun et al. [81] prepared a COF_Pd_ nanocomposite by loading palladium nanoparticles in carboxymethylcellulose-modified COF hydrogel, and the composite showed excellent catalytic performance. Then, folic acid (FA) was modified in COF_Pd_ to construct a signal sensor. The FA-COF_Pd_ sensor can selectively sense folate receptor-positive cancer cells. Through changes in color and fluorescence signals, cancer cells can be accurately and sensitively measured colorimetrically. This method is beneficial to the early diagnosis of cancer cells. Here, the authors used the internal microenvironment of COF to load palladium nanoparticles in situ. This method improved the dispersion of nanozymes and enhanced the stability and enzyme activity of Pd NPs. Similarly, Su et al. [95] prepared Fe^3+^ functionalized COF (Fe-CTF) nanozymes and constructed a colorimetric sensor to detect sarcosine, which is beneficial for the early diagnosis of prostate cancer. Fe-CTF has strong simulated peroxidase activity. Using COF as a carrier, some researchers have introduced smartphones as detectors to improve the detection speed. An electrochemical/visual dual-mode microfluidic device was prepared, and then three-dimensional printing with smartphones was used to quickly detect pheochromocytoma cells (PCC-CTC) [96]. The integrated device utilizes silica-coated magnetic particles to capture PCC-CTC. The COF@Pt nanozyme could catalyze and amplify the signal, detect the signal through electrochemistry, and then use a smartphone app to visually detect the colorimetric signal. This simple, sensitive, and visual platform can detect 2–10^5^ cells/mL PCC-CTC, making the early diagnosis of PCC rapid and accurate. It is expected that these analytical methods will be used as case studies in the future to demonstrate practical application in clinical laboratories.

**Figure 11:**
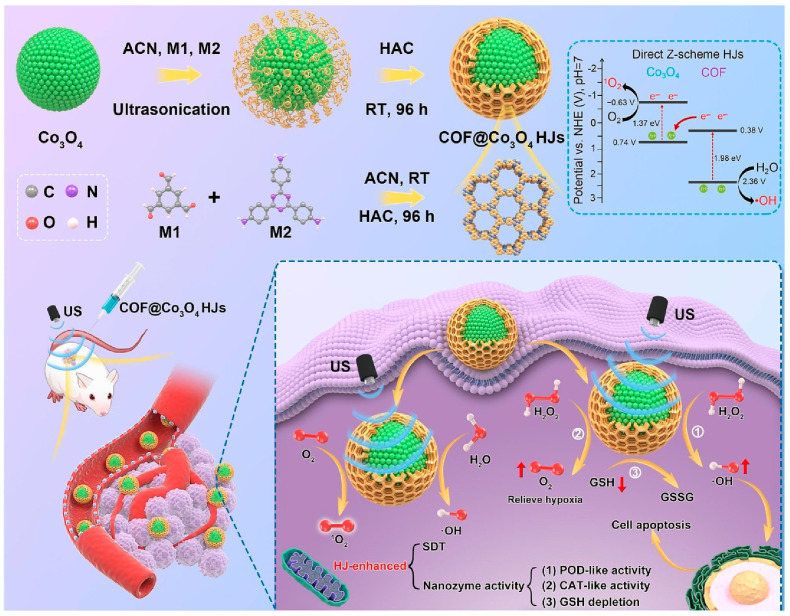
Synthesis and application process of COF@Co_3_O_4_ [79].

Using nanocomplexes as drug carriers and combining them with various disease treatment strategies has aroused great interest among researchers [97]. As a pre-designable porous material, COF is also an ideal drug carrier [98]. The development of COF nanozymes as new disease treatment reactors will be a clinical alternative with great potential. Cancer is a disastrous disease, and current treatments such as chemotherapy are very harmful to the human body. Zhou et al. [30] prepared a multifunctional nanozyme of COF encapsulating AuNPs (COF_Au_) and then encapsulated the cisplatin (C) drug in COF_Au_ to obtain a COF_Au_/C nanozyme. COF_Au_/C has strong glucose oxidase-simulating activity. COFAu/C was incubated with HepG2 cells to obtain a M/COF_Au_/C complex. Under cryo-shock conditions, HepG2 cells lose their proliferation ability and pathogenicity. Tumor cells are ruptured under the action of the laser, and M/COF_Au_/C was cleaved into COF_Au_ and C, thus playing a role in treating liver cancer. Feng et al. [79] loaded COF on the surface of Co_3_O_4_ to prepare a COF@Co_3_O_4_ nanozyme and used it to construct a nanocatalysis–sonodynamic tumor treatment platform. COF@Co_3_O_4_ has strong simulated peroxidase activity. Through the catalytic effect of the COF@Co_3_O_4_ nanozyme, H_2_O_2_ in cells can be quickly converted into O_2_, and reactive oxygen species are increased, effectively achieving the purpose of inhibiting tumors. The experimental principle is shown in Figure 11. Based on the superior performance of COF nanozymes, COF loaded on carbon nanotube (COF-CNT) nanozymes [57] and COF loaded with iodine and ferrocene (TADI-COF-Fc) nanozymes [99] have been used in the treatment of esophageal cancer and other diseases. The MOF@COF nanozyme was developed for enhanced bacterial inhibition [84]. At the same time, Rong et al. [100] also developed a COF nanozyme (COF_FePc_ NSs) with multi-enzyme mimicking activity. COF_FePc_ NSs showed significant therapeutic effects in cancer cells and 4t1 tumor models. In the treatment of diseases, COF drug carriers play an important role and have broad prospects. COFs are used as a kind of nanozyme and are introduced in the treatment plan of diseases, which can expand the contact area and facilitate the loading of drugs. In catalytic therapy, COF nanozymes have high catalytic efficiency, good stability, and good application prospects in the diagnosis and treatment of diseases. However, the biosafety issues of COF nanozymes still need to be paid attention to, including further research on their toxicology. Only on this basis can the practical application of COF nanozymes in clinical experiments be truly considered.

### 3.5. Others

**Figure 12:**
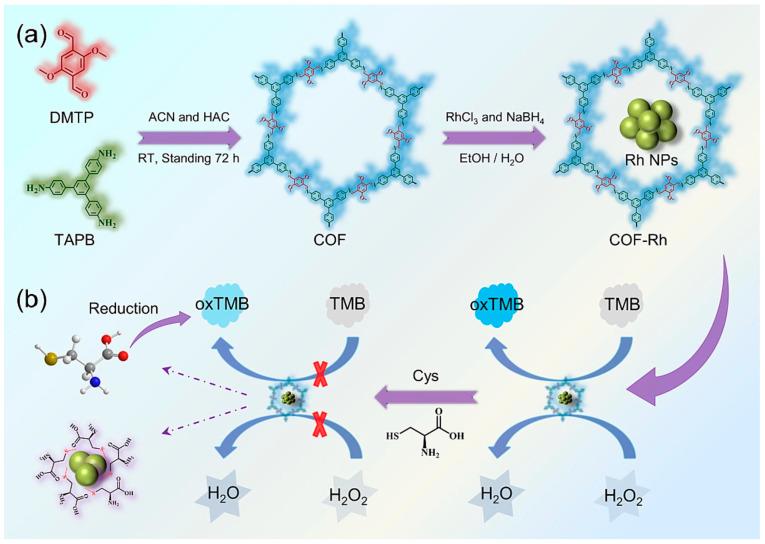
Principles of COF-Rh synthesis (**a**) and Cys detection (**b**) [83].

In addition to the important roles of COF nanozymes in the environment, food, medicine analysis, disease diagnosis, and treatment, their applications in other directions have gradually attracted the attention of researchers. For example, COF nanozyme colorimetric sensors are not uncommon in the detection of biological substances [78,101,102,103,104]. Jin et al. [82] used COF as a carrier to load platinum nanoparticles to prepare COF_Pt_ nanozymes. COF_Pt_ has strong simulated oxidase activity. COF_Pt_ has excellent catalytic activity for TMB oxidation reaction, and glutathione can inhibit this reaction. Therefore, a colorimetric sensor for detecting glutathione content in cells was established. The authors also verified through experiments that COF_Pt_ has good reusability and stability and good practical application prospects. Cysteine is a common amino acid in living organisms. Zhang et al. [83] used COFs as a carrier to load rhodium nanoparticles in situ to prepare a COF-Rh complex. COF-Rh has excellent peroxidase-mimicking activity and can catalyze TMB into blue oxTMB. Cysteine (Cys) can interact with rhodium nanoparticles and reduce the activity of COF-Rh. Through the regulation of TMB oxidation ability by Cys, a colorimetric method was established to detect 0.05–8 μmol/L Cys in serum (Figure 12). In addition, COF-Rh has good stability, which provides a basis for the application of analytical methods. Zhou et al. [105] reported a chiral COF nanozyme. The modification of L-histidine made the nanozyme selective for L-dopa. Some researchers have constructed a COF-Ag nanoenzyme colorimetric platform to detect Hg^2+^ concentration in human blood, which has a good recovery rate. Experimental results show that COF-Ag has strong simulated oxidase activity [73]. A colorimetric sensor constructed based on COF nanozyme to detect 0.27 μmol/L sulfide ions in serum has also been reported [106]. Liang et al. [107] used 3-nitrotyrosine (3-NT) as the analysis object to construct a COF nanozyme fluorescence sensor to detect 3-NT in human serum. The method has strong anti-interference ability. In addition, there are colorimetric sensors based on COF nanozymes for detecting H_2_O_2_, L-arginine, alkaline phosphatase, etc. [108,109,110]. In these reports, COFs usually act as carriers and are important in improving the dispersion and activity of nanozymes. It can be seen that COF nanozymes have great prospects in practical applications. However, before COF nanozymes can be widely used industrially, their stability and reproducibility need to be further studied.

COF nanozymes are an emerging research topic, and their applications in the field of analytical chemistry also show their great potential. At present, COF nanozymes mainly simulate oxidases and peroxidases. Many methods for determining analytes have been developed based on COF nanozymes, among which colorimetric methods are the main ones. With the advancement of modern technology, some researchers have designed smartphone detection applets and microfluidic devices and introduced them into the application of COF nanozymes, creating new detection technology. It can be seen that combining modern technology with COF nanozymes to develop intelligent monitoring equipment to detect analytes may become a trend in the future. Through the summary of analysis methods, it can also be found that the catalytic properties of COF nanozymes play a greater role in improving the analysis signal. Among the currently reported analytical methods, hybrid COF nanozymes are widely used, and the special structure of COF has become an excellent cultivation soil for hybrids. However, compared with other nanozymes, the types and number of targets that can be analyzed by COF nanozyme-based analysis methods are still extremely limited. At the same time, the selectivity of COF nanozymes cannot be guaranteed during the construction of analytical methods. Some researchers have improved the selectivity of COF nanozymes by combining single-stranded DNA, oligopeptides, or molecularly imprinted polymers. Judging from the current research progress, although COF nanozymes still face great challenges, their enzyme-like catalytic properties and versatility are attracting more and more attention. It is believed that in the next few decades, a series of analytical methods based on COF nanozymes will appear, expanding their application in analytical chemistry.

## 4. Potential Catalytic Mechanism of COF Nanozymes

Research on catalytic mechanisms is of great significance to the development of new COF nanozymes and the regulation of their catalytic activity. There are many common types of mimetic enzymes. The currently reported COF nanozymes are mainly oxidation mimetic enzymes and peroxide mimetic enzymes. There are relatively few reports on other mimetic enzymes. Since the report of COF nanozymes, researchers have been committed to studying the catalytic mechanism of COF nanozymes [111]. Zhang et al. [40] reported a COF nanozyme (Tph-BDP) that simulates oxidase activity. Under light conditions, TMB is catalytically oxidized by H_2_O_2_ to generate a blue product. Both free radical capture experiments and electron paramagnetic resonance (EPR) spectra proved the existence of the intermediate product O_2_^·−^. Calculated from the Michaelis–Menten equation, the *K*_m_ value of Tph-BDP is 98.15 × 10^−6^ mol/L, indicating that it has a strong affinity for TMB. In addition, the formation of Tph-BDP is conducive to rapid charge transfer and accelerates the reaction rate. Similar catalytic mechanisms have also been reported in other references [71,78]. In addition to oxidation mimetic enzymes, researchers have also reported the catalytic mechanism of COF nanozymes that imitate peroxidase. Hou et al. [62] developed a hybrid COF nanozyme (Fe_3_O_4_@ZIF-8@COF) with simulated peroxidase activity. In the presence of H_2_O_2_, the substrate 4-aminoacylantipyrine is catalytically oxidized to generate the intermediate substance •OH. The reaction also follows the Michaelis–Menten kinetic equation. It can be seen from the results that the *K*_m_ value of Fe_3_O_4_@ZIF-8@COF is lower than that of HRP, indicating that Fe_3_O_4_@ZIF-8@COF has a stronger affinity for the substrate. This may be due to the porous structure of COF, which facilitates the adsorption of substrates, thereby accelerating the catalytic rate. Similar catalytic mechanisms of COF nanozymes that mimic peroxidase have also been reported in other references. [57] From the current research situation, the capture of intermediate products is one of the important means to study the activity of simulated enzymes.

In order to conduct a more in-depth exploration of the potential mechanism behind the catalytic activity of COF nanozymes, researchers introduced density functional theory (DFT) calculations based on the above studies. Li et al. [106] synthesized a COF nanozyme with oxidase-like activity (ETTA-Tz COF) and constructed a colorimetric probe to detect sulfide ions. The authors demonstrated through various characterization methods that ETTA-Tz COF has low charge transfer resistance, which is conducive to the improvement of photoelectric properties. In addition, free radical scavenging experiments and EPR experiments also showed that O_2_^·−^ and h^+^ are the main reaction intermediates catalyzed by the ETTA-Tz COF nanozyme. On this basis, the authors used DFT calculations to study the influence of electron acceptors (monomers) on the band gap and charge behavior. The calculation results were then used to optimize the structure of the COF nanozyme, and the corresponding HOMO and LUMO molecular models were constructed. Based on the importance of DFT calculation, it has gradually attracted the attention of researchers in the study of the catalytic mechanism of COF nanozymes [42,76,77]. In the study of these potential catalytic mechanisms, the introduction of DFT calculations has important guiding significance for the design and synthesis of COF nanozymes. However, judging from the current reports, most applications of COF nanozymes are still focused on the research stage of catalytic activity, and the research on the catalytic mechanism is still relatively superficial. Some references do not even involve the study of the mechanism. Research on theoretical calculations and molecular modeling needs to be further strengthened. This is also one of the future research directions of COF nanozymes.

## 5. Conclusions and Perspectives

Since the report on COF nanozymes, researchers have made significant efforts to research the types and preparation methods of COF nanozymes. These outstanding contributions provide a potential platform for the application of COF nanozymes. Recently, significant progress has been made using COF-based nanomaterials to mimic natural enzymes. Although the application of COF nanozymes in the analytical field has just begun to be studied, it still shows great application potential due to its controllable skeleton structure and excellent catalytic performance. In this review, we discuss COF nanozymes, focusing on their applications in environmental, food, and pharmaceutical analyses, as well as in disease diagnosis and treatment.

Although COF nanozymes offer huge scope for the analytical field, there are still some challenges that need to be overcome. First, the introduction of COFs improves the dispersion and stability of nanoparticles and shows similar catalytic capabilities to natural enzymes. However, COF nanozymes obtained by combining the excellent functions of COFs and nanomaterials may lead to a decrease in enzyme activity. Making these COF-loaded nanoparticles exhibit uniform size and long-term stability while also possessing excellent enzymatic activity still requires further research.

Second, although COF nanozymes have shown excellent potential in the environment, food, medicine, and disease diagnosis and treatment, they still face the problems of unclear structure–activity relationships and unclear catalytic mechanisms. How the reaction process, mechanism, and catalytic kinetics through theoretical calculations and model construction can be thoroughly studied deserves in-depth consideration.

Third, although the concept of COF nanozymes has been around for many years, its application scope is relatively limited. So far, the reported COF nanozymes are mainly oxidation and peroxide mimetic enzymes, and there are few reports on other mimetic enzymes. In addition, although COF nanozymes have been developed in multiple analytical fields, these reports have not yet formed a systematic study, especially since there are few reports related to the analysis of complex samples. The expansion of the application range of COF nanozymes and solving these foreseeable challenges require attention and solution strategies.

Fourth, although COF nanozymes have superior performance, the ethical and social impacts (including issues such as biosafety, environmental impact, and potential regulatory frameworks) caused by their widespread use have not attracted enough attention. Solving the above problems through toxicological research on COF nanozymes should be paid attention to by everyone.

Although there are still many problems that need to be overcome in the research of COF nanozymes, there is no doubt about their development potential. Below, we briefly outline the potential future development directions of COF nanozymes. (1) Explore new COF nanozymes. Compared with natural enzymes, COF nanozymes have poor catalytic activity and selectivity. Based on the designability of the COF structure, it is an interesting research direction to combine chiral substances, DNA, molecularly imprinted polymers, nanozymes, and COF to prepare highly active new composite COF nanozymes. (2) Expansion of COF nanozyme functions. At present, the main application function of COF nanozymes is catalytic signal amplification, which has a good effect on improving the sensitivity of analytical methods. In addition to their catalytic functions, COF nanozymes can also combine external energy sources such as sound, heat, electricity, and magnetism to fully utilize their combined effects to develop COF nanozymes with special functions, such as sonosensitizers. (3) Research on new sensing technology of COF nanozymes. At present, although various analysis methods based on COF nanozymes have been reported, the types and numbers of detection targets are still relatively small. With the advancement of modern science and technology, the development of new sensing technologies by combining COF nanozymes with advanced technologies (such as artificial intelligence, microfluidic devices, etc.) will become the next research hotspot. (4) Study on the catalytic mechanism of COF nanozymes. The catalytic mechanism is of great significance to the design and synthesis of COF nanozymes. However, there are relatively few studies on the catalytic mechanism of COF nanozymes, and the application of theoretical calculations and molecular modeling in the study of catalytic mechanisms is even more lacking. Therefore, the study of the catalytic mechanism of COF nanozymes will be a research direction that cannot be ignored.

As an emerging research direction in recent years, COF nanozymes have only summarized the preliminary status of their application in the analytical field. It is believed that shortly, with the further maturity of COF nanozyme design and synthesis technology, the simple, sensitive, and accurate COF nanozyme catalytic system will shine brilliantly in promoting the analytical field.

## Figures and Tables

**Table 1:** Performance comparison of COF nanozymes.

Type of COF Nanozyme	COF Nanozyme	Type of COF	Loaded Particles	Load Way	Synthesis Method	Temperature (°C)/Time	Type of Mimic Enzyme	Characteristics	Reference
Unloaded COF nanozymes	TTA-Tp COF	Triazine-based COF	——	——	Solvothermal synthesis	120 °C/72 h	Mimic oxidase	Excellent catalytic ability, good stability	[36]
	Tph-BDP	Imine-linked COF			Solvothermal synthesis	120 °C/120 h	Mimic oxidase	Sensitive and selective	[40]
	DAFB-DCTP COF	Vinylidene-linked COF			Solvothermal synthesis	180 °C/72 h	Mimic hydrolase	Reliability and simplicity	[42]
	CTF-1	Triazine-based COF			Microwave-assisted synthesis	—/30 min	Mimic oxidase and peroxidase	Low cost, easy preparation	[72]
	Tph-BT	Imine-linked COF			Solvothermal synthesis	150 °C/72 h	Mimic oxidase and peroxidase	Sensitive and selective	[76]
	TAS-COF	Imine-linked COF			Solvothermal synthesis	120 °C/72 h	Mimic oxidase	Simple and fast	[77]
	COF-300-AR	Imine-linked COF			NaBH_4_ -reducing method	0 °C/1 h	Mimic oxidase	Easy, light control and good reusability	[78]
Metal-loaded COF nanozymes	TPE-s COF-Au@Cisplatin	Imine-linked COF	AuNPs	Au-SH bond	NaBH_4_-reducing method	0 °C/8 h	Mimic glucose oxidase	Facile	[30]
	COF-AuNPs	Imine-linked COF	AuNPs	Coordination of unsaturated amino groups	Citrate-reducing method	Room temperature/-	Mimic peroxidase	Good accuracy and sensitivity	[53]
	Cu-Cys@COF-OMe	Imine-linked COF	Cu^+^/Cu^2+^	Cu-coordination bond	Coordination	150 °C/5 h	Mimic laccase	Excellent stability	[54]
	COF@Co_3_O_4_	Imine-linked COF	Co_3_O_4_	Electrostatic force	Sonochemical synthesis	Room temperature/96 h	Mimic peroxidase, catalase, and glutathione peroxidase	Complete eradication of tumors	[79]
	PB@Fe-COF@Au	Imine-linked COF	AuNPs	Au–SH bond	Citrate-reducing method	Room temperature/30 min	Mimic peroxidase	Affordable, sensitive, and selective	[80]
	PdNPs/CMC-COF-LZU1	Imine-linked COF	PdNPs	In situ coordination	Reflux	78 °C/3 h	——	Accurate and sensitive	[81]
	Pt NPs/COF-300-AR	Imine-linked COF	PtNPs	Nitrogen atom affinity	NaBH_4_-reducing method	Room temperature/3 h	Mimic oxidase	Good stability and high reusability	[82]
	COF-Rh	Imine-linked COF	RhNPs	Electrostatic force	NaBH_4_-reducing method	Room temperature/15 h	Mimic peroxidase	Good catalytic properties	[83]
Other COF nanozymes	GOD-MP-11/COF_ETTA-TPAL_	Imine-linked COF	Glucose oxidase and microperoxidase-11	Hydrogen bonds	Doping	4 °C/10 h	Mimic glucose oxidase and microperoxidase-11	Large surface area	[55]
	COF-CNT	Imine-linked COF	Nanotube	Doping	Solvothermal synthesis	70 °C/24 h	Mimic peroxidase	Good catalytic properties	[57]
	MOFs-COFs	Imine-linked COF	MOFs	Affinity	Solvothermal synthesis	80 °C/12 h	Mimic peroxidase	High selectivity and excellent stability	[62]
	MOF@COF	Imine-linked COF	MOFs	Schiff base reaction	Solvothermal synthesis	110 °C/48 h	Mimic peroxidase	Good treatment effect	[84]
	hCOF	Imine-linked COF	ZIF-8	π-stacking	Room temperature etching	Room temperature/1 h	Mimic oxidase	Good catalytic effect	[85]
	COF-300-AR@CRL	Imine-linked COF	Candida rugosa lipase	Electrostatic force	Self-assembly strategy	Room temperature/12 h	Mimic oxidase	Dual enzymatic catalytic activities	[86]

## Data Availability

Data sharing not applicable.
